# Supplementary material for: A multicenter clinical AI system study for detection and diagnosis of focal liver lesions
Source: Nat Commun. 2024 Feb 7;15:1131. doi: 10.1038/s41467-024-45325-9 (PMC10850133; doi:10.1038/s41467-024-45325-9)
Supplement: Supplementary file 1 — Supplementary Information [file 41467_2024_45325_MOESM1_ESM.pdf]

Supplement data

Number of figures and tables: **3 figures and 7 tables**

Figure S1: Comparative Analysis of Diagnostic Performance Between Models Trained with Diverse Training Sets (Single-center vs Multi-center): (a) A seven-category classification comparison based on AUC, accuracy, sensitivity, specificity, and precision within the ZZH cohort; (b) A similar comparison in the QZH cohort; (c) A similar comparison in the NBH cohort. 'Source data are provided as a Source Data file (Source_data_Figure_S1.xlsx).'

Figure S2: Comparative Evaluation of Diagnostic Performance Among Different Models (Trained with and without Phase Interaction): (a) A seven-category classification comparison based on AUC, accuracy, sensitivity, specificity, and precision within the ZZH cohort; (b) A similar comparison in the QZH cohort; (c) A similar comparison in the NBH cohort. 'Source data are provided as a Source Data file (Source_data_Figure_S2.xlsx).'

Figure S3: Overview of Data and Cohort: (a) Distribution and statistics of CT imaging device manufacturers across 18 data centers; (b) Patient demographics and lesion distribution within the training, internal, external, and prospective cohorts. 'Source data are provided as a Source Data file (Source_data_Figure_S3.xlsx).'

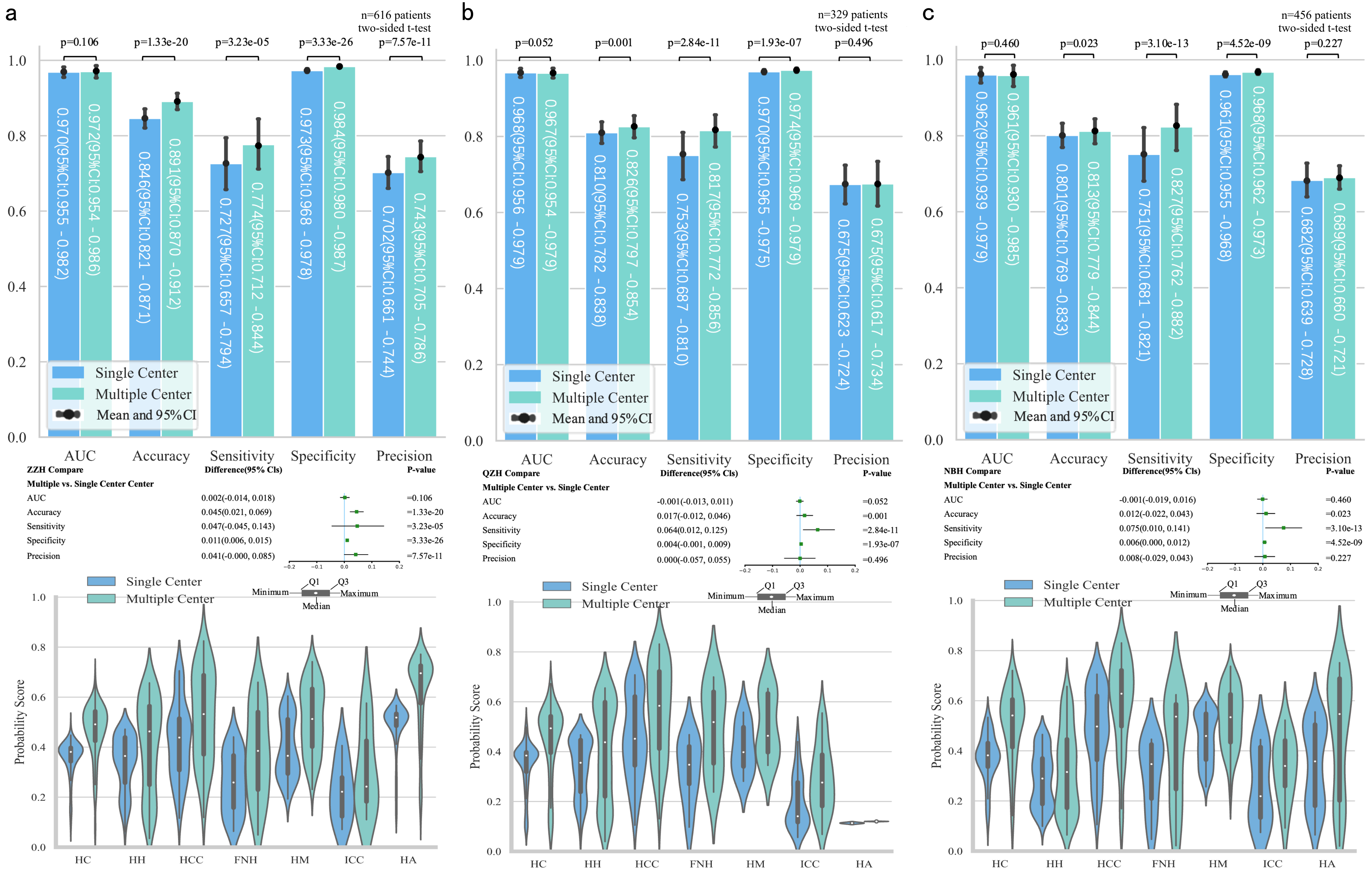

ZZH=Zhangzhou Hospital; QZH=Quzhou People's Hospital; NBH=Ningbo No.2 Hospital; AUC=Area Under Curve; 95%CI = 95% Confidence Interval; Q1=Lower Quartile; Q3=Upper Quartile; HCC=Hepatocellular Carcinoma; ICC=Intrahepatic Cholangiocarcinoma; HM=Hepatic Metastasis; HC=Hepatic Cyst; HH=Hepatic Haemangioma; FNH=Focal Nodular Hyperplasia; HA=Hepatic Abscess.

Figure S1: Comparative Analysis of Diagnostic Performance Between Models Trained with Diverse Training Sets (Single-center vs Multi-center): (a) A seven-category classification comparison based on AUC, accuracy, sensitivity, specificity, and precision within the ZZH cohort; (b) A similar comparison in the QZH cohort; (c) A similar comparison in the NBH cohort.

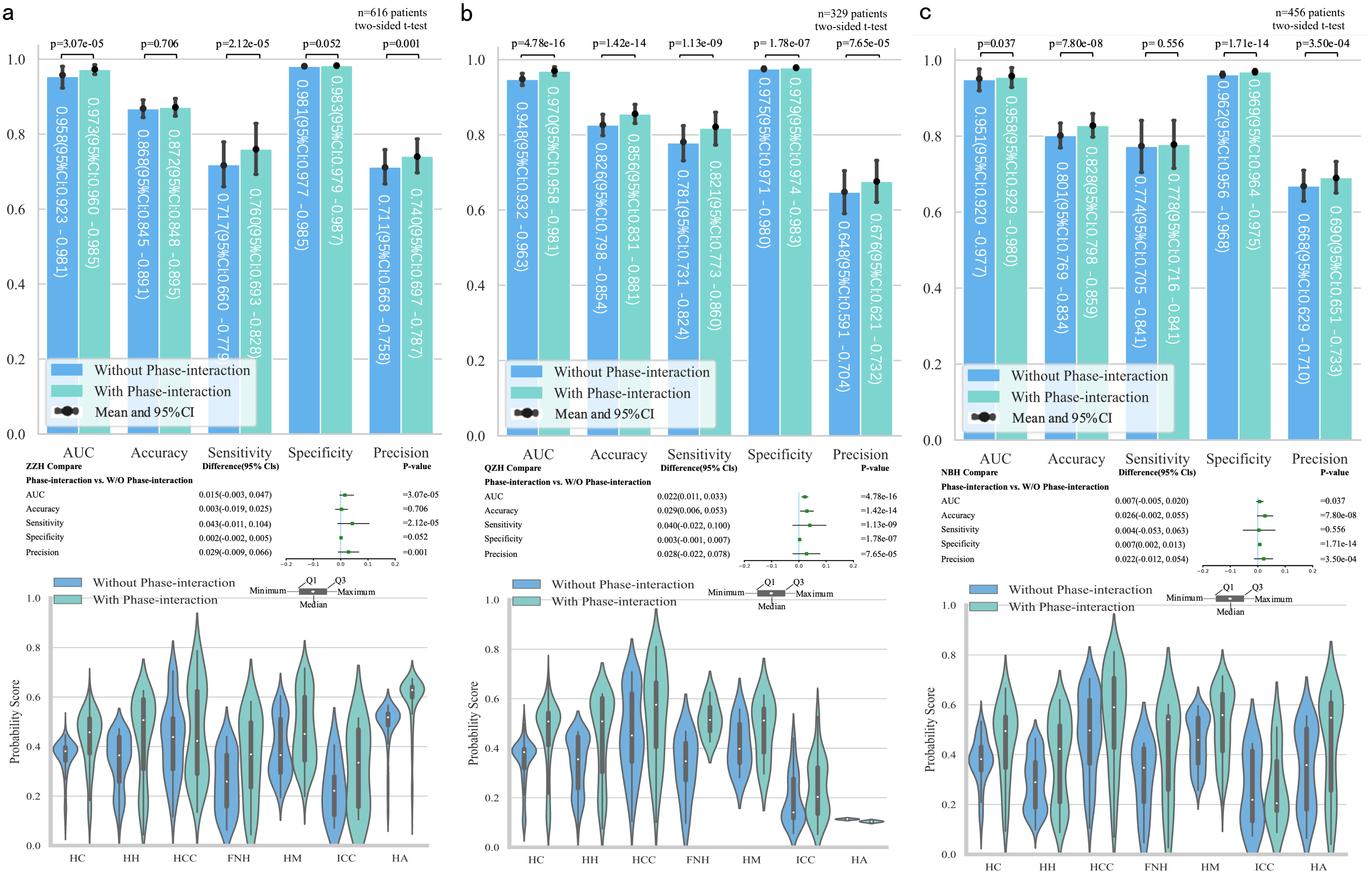

ZZH=Zhangzhou Hospital; QZH=Quzhou People's Hospital; NBH=Ningbo No.2 Hospital; AUC=Area Under Curve; 95%CI = 95% Confidence Interval; Q1=Lower Quartile; Q3=Upper Quartile; HCC=Hepatocellular Carcinoma; ICC=Intrahepatic Cholangiocarcinoma; HM=Hepatic Metastasis; HC=Hepatic Cyst; HH=Hepatic Haemangioma; FNH=Focal Nodular Hyperplasia; HA=Hepatic Abscess.

Figure S2: Comparative Evaluation of Diagnostic Performance Among Different Models (Trained with and without Phase Interaction): (a) A seven-category classification comparison based on AUC, accuracy, sensitivity, specificity, and precision within the ZZH cohort; (b) A similar comparison in the QZH cohort; (c) A similar comparison in the NBH cohort.

a

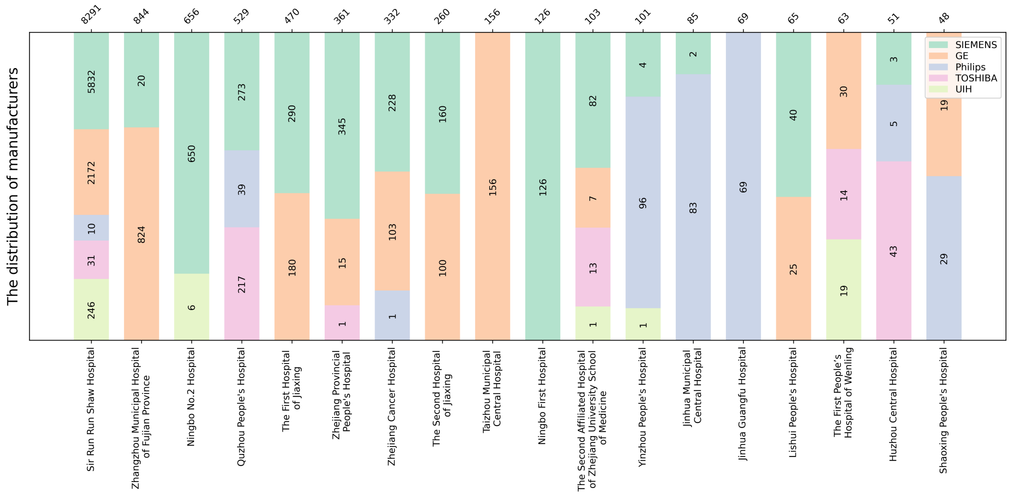

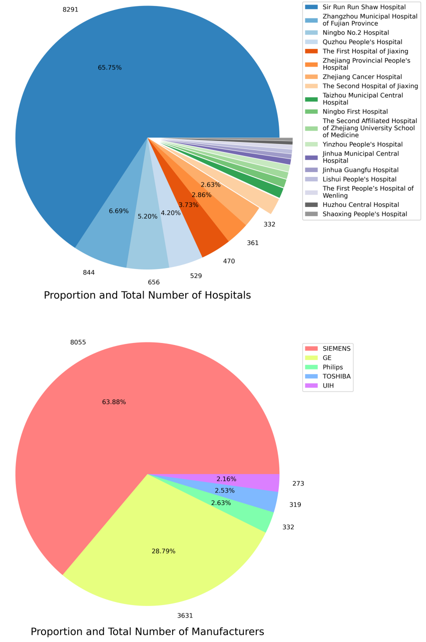

GE: General Electrics; UIE:United Imaging Healthcare.

b

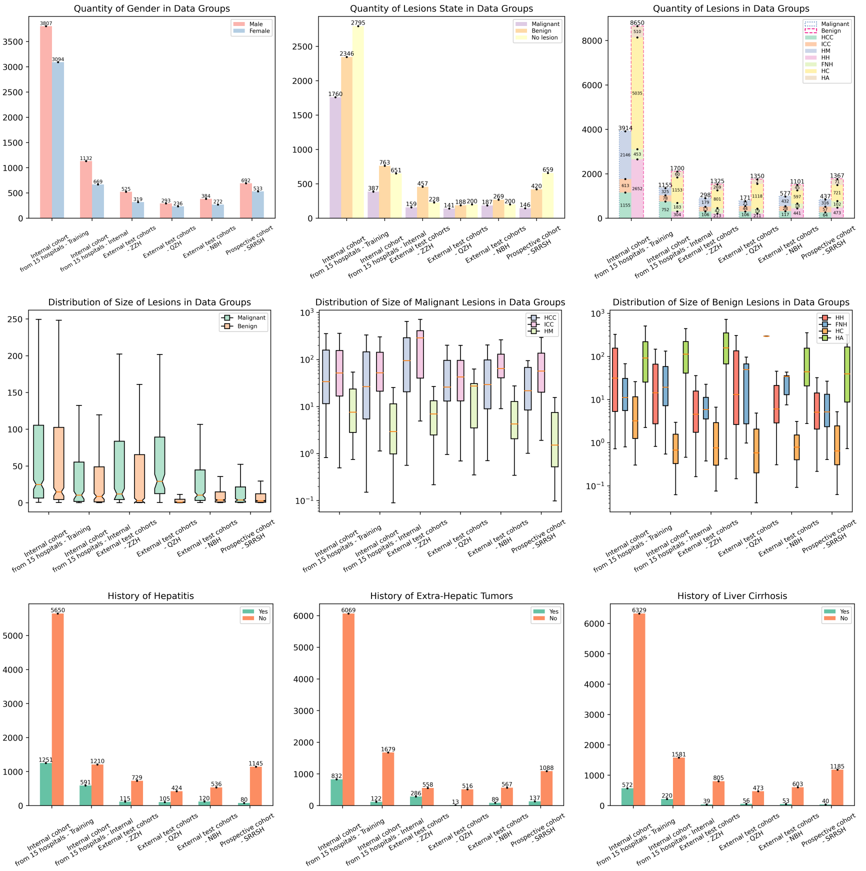

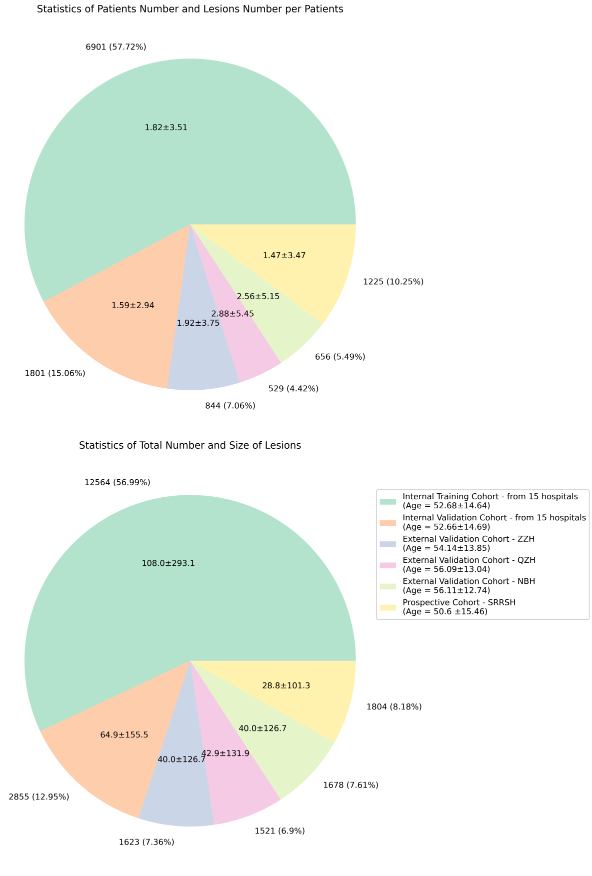

HCC: Hepatocellular Carcinoma; ICC: Intrahepatic Cholangiocarcinoma; HM: Hepatic Metastasis; HC: Hepatic Cyst; HH: Hepatic Haemangioma; FNH: Focal Nodular Hyperplasia; HA: Hepatic Abscess.

ZZH=Zhangzhou Hospital; QZH=Quzhou People's Hospital; NBH=Ningbo No.2 Hospital.

Figure S3: Overview of Data and Cohort: (a) Distribution and statistics of CT imaging device manufacturers across 18 data centers; (b) Patient demographics and lesion distribution within the training, internal, external, and prospective cohorts.

Supplementary Tables

Table S1: The performance of LiAIDS for seven-category classification.

		Internal validation	External validations			Prospective validation
			ZZH	QZH	NBH	
HCC	Sensitivity	0.986(95CI0.955-1.000)	0.758(95CI0.667-0.840)	0.918(95CI0.855-0.967)	0.917(95CI0.864-0.963)	0.878(95CI0.771-0.971)
	Specificity	0.960(95CI0.940-0.980)	0.986(95CI0.976-0.994)	0.955(95CI0.939-0.972)	0.965(95CI0.948-0.981)	0.956(95CI0.942-0.971)
	Precision	0.835(95CI0.753-0.916)	0.882(95CI0.810-0.948)	0.774(95CI0.700-0.849)	0.866(95CI0.802-0.923)	0.522(95CI0.406-0.639)
ICC	Sensitivity	0.618(95CI0.440-0.774)	0.444(95CI0.125-0.818)	0.375(95CI0.229-0.531)	0.417(95CI0.125-0.727)	0.405(95CI0.262-0.542)
	Specificity	0.992(95CI0.982-1.000)	0.980(95CI0.970-0.989)	0.973(95CI0.960-0.986)	0.975(95CI0.961-0.987)	0.992(95CI0.985-0.997)
	Precision	0.875(95CI0.731-1.000)	0.200(95CI0.048-0.391)	0.469(95CI0.290-0.645)	0.250(95CI0.067-0.467)	0.769(95CI0.591-0.926)
HM	Sensitivity	0.865(95CI0.778-0.937)	0.897(95CI0.838-0.948)	0.900(95CI0.667-1.000)	0.941(95CI0.901-0.977)	0.939(95CI0.892-0.981)
	Specificity	0.980(95CI0.965-0.994)	0.977(95CI0.965-0.987)	0.964(95CI0.949-0.978)	0.955(95CI0.936-0.973)	0.931(95CI0.912-0.950)
	Precision	0.901(95CI0.825-0.966)	0.867(95CI0.800-0.924)	0.273(95CI0.133-0.444)	0.858(95CI0.803-0.913)	0.698(95CI0.628-0.767)
HH	Sensitivity	0.912(95CI0.806-1.000)	0.777(95CI0.703-0.846)	0.837(95CI0.774-0.892)	0.731(95CI0.670-0.788)	0.756(95CI0.708-0.802)
	Specificity	0.964(95CI0.944-0.982)	0.983(95CI0.973-0.992)	0.992(95CI0.984-0.998)	0.990(95CI0.979-0.998)	0.985(95CI0.973-0.996)
	Precision	0.689(95CI0.540-0.824)	0.908(95CI0.854-0.958)	0.970(95CI0.938-0.993)	0.976(95CI0.949-0.994)	0.973(95CI0.951-0.992)
FNH	Sensitivity	0.869(95CI0.807-0.925)	0.781(95CI0.632-0.919)	1.000(95CI1.000-1.000)	0.786(95CI0.538-1.000)	0.901(95CI0.825-0.966)
	Specificity	0.990(95CI0.976-1.000)	0.979(95CI0.968-0.988)	0.987(95CI0.978-0.994)	0.935(95CI0.915-0.955)	0.942(95CI0.924-0.959)
	Precision	0.975(95CI0.945-1.000)	0.610(95CI0.457-0.763)	0.571(95CI0.350-0.778)	0.224(95CI0.113-0.346)	0.599(95CI0.505-0.691)
HC	Sensitivity	0.935(95CI0.833-1.000)	0.904(95CI0.862-0.945)	0.899(95CI0.866-0.929)	0.879(95CI0.800-0.952)	0.857(95CI0.926-0.914)
	Specificity	0.987(95CI0.975-0.997)	0.993(95CI0.987-0.998)	1.000(95CI1.000-1.000)	0.996(95CI0.991-1.000)	0.994(95CI0.988-0.999)
	Precision	0.853(95CI0.720-0.967)	0.977(95CI0.951-0.995)	1.000(95CI1.000-1.000)	0.967(95CI0.917-1.000)	0.964(95CI0.926-0.992)
HA	Sensitivity	0.907(95CI0.810-0.979)	0.977(95CI0.954-0.995)	0.000(95CI0.000-0.000)	0.757(95CI0.606-0.889)	0.861(95CI0.770-0.940)
	Specificity	0.987(95CI0.975-0.997)	0.950(95CI0.932-0.968)	0.973(95CI0.962-0.985)	0.988(95CI0.977-0.996)	0.978(95CI0.966-0.988)
	Precision	0.886(95CI0.784-0.976)	0.879(95CI0.836-0.919)	0.000(95CI0.000-0.000)	0.800(95CI0.667-0.923)	0.773(95CI0.667-0.871)

LiAIDS=Liver Artificial Intelligence Diagnosis System;

ZZH=Zhangzhou Hospital; QZH=Quzhou People's Hospital; NBH=Ningbo No.2 Hospital;

HCC=Hepatocellular Carcinoma; ICC=Intrahepatic Cholangiocarcinoma; HM= Hepatic Metastasis; HH= Hepatic Haemangioma; FNH= Focal Nodular Hyperplasia; HC= Hepatic Cyst; HA= Hepatic Abscess.

Table S2: The performance of LiAIDS and radiologists for seven-category classification in the comparative study.

		LiAIDS	Junior 1	Junior 2	Junior 3	Senior 1	Senior 2	Senior3	Junior 1 + LiAIDS	Junior 2 + LiAIDS	Junior 3 + LiAIDS	Senior 1 + LiAIDS	Senior 2 + LiAIDS	Senior 3 + LiAIDS
HCC	Sensitivity	0.882(0.767-0.974)	0.412	0.457	0.571	0.686	0.706	0.829	0.903	0.806	0.710	0.871	0.871	0.833
	Specificity	0.972(0.957-0.984)	0.942	1.000	0.993	0.999	0.982	0.996	0.988	0.997	0.992	0.997	0.990	0.997
	Precision	0.638(0.500-0.778)	0.246	1.000	0.800	0.960	0.667	0.906	0.800	0.926	0.815	0.931	0.818	0.926
ICC	Sensitivity	0.400(0.100-0.750)	0.100	0.222	0.778	0.333	0.444	0.222	0.647	0.200	0.412	0.333	0.118	0.313
	Specificity	0.994(0.987-0.998)	0.986	0.997	0.954	0.997	0.986	1.000	0.995	0.998	0.993	0.998	0.998	1.000
	Precision	0.500(0.143-0.875)	0.091	0.500	0.171	0.600	0.286	1.000	0.786	0.750	0.636	0.857	0.667	1.000
HM	Sensitivity	0.889(0.773-0.974)	0.385	0.500	0.366	0.718	0.730	0.756	0.912	0.909	0.909	0.912	0.824	0.882
	Specificity	0.932(0.913-0.952)	0.997	0.996	0.974	0.991	0.968	0.996	0.973	0.973	0.957	0.982	0.993	0.978
	Precision	0.438(0.324-0.556)	0.882	0.870	0.455	0.824	0.551	0.912	0.660	0.652	0.536	0.738	0.875	0.698
HH	Sensitivity	0.761(0.715-0.808)	0.896	0.983	0.975	0.986	0.885	0.992	0.926	0.952	0.945	0.955	0.965	0.971
	Specificity	0.987(0.974-0.997)	0.814	0.783	0.810	0.903	0.983	0.905	0.959	0.956	0.987	0.990	0.959	0.965
	Precision	0.984(0.967-0.996)	0.796	0.777	0.803	0.900	0.981	0.903	0.957	0.955	0.986	0.990	0.959	0.965
FNH	Sensitivity	0.897(0.817-0.962)	0.121	0.144	0.221	0.727	0.899	0.609	0.774	0.820	0.903	0.967	0.823	0.823
	Specificity	0.934(0.913-0.953)	0.998	0.992	0.998	0.994	0.968	0.998	0.991	0.988	0.982	0.991	0.993	0.998
	Precision	0.616(0.517-0.711)	0.917	0.722	0.950	0.941	0.800	0.981	0.906	0.877	0.848	0.922	0.927	0.981
HC	Sensitivity	0.896(0.833-0.951)	0.956	0.982	0.866	0.938	0.934	0.964	0.880	0.981	0.981	0.991	1.000	0.954
	Specificity	0.994(0.987-1.000)	0.911	0.973	0.967	0.979	0.990	0.979	0.981	0.977	0.981	0.966	0.957	0.986
	Precision	0.972(0.937-1.000)	0.639	0.865	0.822	0.890	0.943	0.893	0.905	0.898	0.914	0.859	0.824	0.937
HA	Sensitivity	0.873(0.776-0.953)	0.642	0.500	0.582	0.949	0.873	0.963	0.927	0.852	0.782	0.893	0.873	1.000
	Specificity	0.983(0.972-0.993)	0.994	0.996	0.995	0.995	0.976	0.986	0.979	0.991	1.000	0.996	0.995	0.981
	Precision	0.828(0.722-0.923)	0.929	0.890	0.939	0.961	0.821	0.895	0.810	0.902	1.000	0.962	0.941	0.831

LiAIDS= Liver Artificial Intelligence Diagnosis System；

HCC=Hepatocellular Carcinoma; ICC=Intrahepatic Cholangiocarcinoma; HM= Hepatic Metastasis; HH= Hepatic Haemangioma; FNH= Focal Nodular Hyperplasia; HC= Hepatic Cyst; HA= Hepatic Abscess.

Table S3: Comparison of LiAIDS and the models with clinical data only or image data only.

		Clinical Data Only	Image Data Only	Image + Clinical Data (LiAIDS)
Internal validation	AUC (95%CI)	0.801 (0.776-0.827)	0.949 (0.938-0.960)	0.982 (0.974-0.989)
	Accuracy (95%CI)	0.649 (0.605-0.694)	0.786 (0.748-0.824)	0.880 (0.847-0.911)
	Precision (95%CI)	0.466 (0.409-0.532)	0.753 (0.700-0.803)	0.855 (0.811-0.895)
	Sensitivity (95%CI)	0.440 (0.419-0.461)	0.769 (0.720-0.814)	0.864 (0.825-0.901)
	Specificity (95%CI)	0.976 (0.969-0.982)	0.963 (0.955-0.971)	0.979 (0.973-0.985)
ZZH	AUC (95%CI)	0.734 (0.687-0.779)	0.952 (0.932-0.968)	0.973 (0.960-0.985)
	Accuracy (95%CI)	0.484 (0.450-0.519)	0.794 (0.766-0.822)	0.872 (0.848-0.895)
	Precision (95%CI)	0.350 (0.315-0.387)	0.667 (0.624-0.712)	0.740 (0.697-0.787)
	Sensitivity (95%CI)	0.372 (0.339-0.406)	0.702(0.635-0.768)	0.760 (0.693-0.828)
	Specificity (95%CI)	0.967 (0.962-0.971)	0.974(0.969-0.978)	0.983 (0.979-0.987)
QZH	AUC (95%CI)	0.779 (0.757-0.802)	0.948(0.929-0.965)	0.970 (0.958-0.981)
	Accuracy (95%CI)	0.167 (0.140-0.194)	0.809(0.778-0.839)	0.856 (0.831-0.881)
	Precision (95%CI)	0.390 (0.327-0.449)	0.656(0.608-0.705)	0.676 (0.621-0.732)
	Sensitivity (95%CI)	0.456 (0.413-0.491)	0.752(0.690-0.810)	0.821 (0.773-0.860)
	Specificity (95%CI)	0.975 (0.970-0.980)	0.972(0.967-0.977)	0.979 (0.974-0.983)
NBH	AUC (95%CI)	0.818 (0.778-0.853)	0.923(0.896-0.944)	0.958 (0.929-0.980)
	Accuracy (95%CI)	0.489 (0.449-0.529)	0.701(0.665-0.736)	0.828 (0.798-0.859)
	Precision (95%CI)	0.480 (0.437-0.521)	0.612(0.578-0.646)	0.690 (0.651-0.733)
	Sensitivity (95%CI)	0.438 (0.395-0.477)	0.697(0.632-0.757)	0.778 (0.716-0.841)
	Specificity (95%CI)	0.980 (0.976-0.985)	0.949(0.942-0.955)	0.969 (0.964-0.975)
Prospective cohort	AUC (95%CI)	0.763 (0.744-0.782)	0.956 (0.945-0.967)	0.967 (0.956-0.977)
	Accuracy (95%CI)	0.318 (0.287-0.351)	0.768 (0.740-0.797)	0.805 (0.777-0.833)
	Precision (95%CI)	0.338 (0.294-0.380)	0.700 (0.658-0.741)	0.754 (0.713-0.794)
	Sensitivity (95%CI)	0.396 (0.367-0.424)	0.726 (0.688-0.764)	0.789 (0.755-0.822)
	Specificity (95%CI)	0.952 (0.946-0.958)	0.960 (0.954-0.965)	0.967 (0.961-0.972)

LiAIDS: Liver Artificial Intelligence Diagnosis System；

ZZH=Zhangzhou Hospital; QZH=Quzhou People's Hospital; NBH=Ningbo No.2 Hospital.

Table S4: Performance comparison using different training datasets (single center vs multi-center).

	Training using data from a single center (3,167 cases from SRRSH)			Training using data from multiple centers (3,167 cases from 15 hospitals)		
	ZZH	QZH	NBH	ZZH	QZH	NBH
Binary						
AUC (95%CI)	0.983 (0.975-0.989)	0.971 (0.955-0.984)	0.982 (0.974-0.989)	0.990 (0.985-0.994)	0.983 (0.974-0.991)	0.982 (0.973-0.990)
Accuracy (95%CI)	0.916 (0.896-0.935)	0.892 (0.869-0.915)	0.897 (0.872-0.920)	0.939 (0.922-0.955)	0.901 (0.879-0.923)	0.907 (0.884-0.930)
Precision (95%CI)	0.808 (0.758-0.857)	0.693 (0.628-0.757)	0.834 (0.792-0.874)	0.857 (0.813-0.900)	0.696 (0.634-0.756)	0.837 (0.796-0.878)
Sensitivity (95%CI)	0.920 (0.881-0.952)	0.905 (0.855-0.951)	0.959 (0.934-0.981)	0.938 (0.904-0.967)	0.966 (0.933-0.993)	0.981 (0.964-0.996)
Specificity (95%CI)	0.915 (0.890-0.938)	0.889 (0.864-0.916)	0.848 (0.808-0.884)	0.939 (0.919-0.958)	0.883 (0.858-0.910)	0.848 (0.810-0.887)
Seven-category						
AUC (95%CI)	0.970 (0.955-0.982)	0.968 (0.956-0.979)	0.962 (0.939-0.979)	0.972 (0.954-0.986)	0.967 (0.954-0.979)	0.961 (0.930-0.985)
Accuracy (95%CI)	0.846 (0.821-0.871)	0.810 (0.782-0.838)	0.801 (0.769-0.833)	0.891 (0.870-0.912)	0.826 (0.797-0.854)	0.813 (0.779-0.844)

LiAIDS = Liver Artificial Intelligence Diagnosis System.

AUC=area under the receiver operating characteristic curve.

SRRSH= Sir Run Run Shaw Hospital; ZZH=Zhangzhou Hospital; QZH=Quzhou People's Hospital; NBH=Ningbo No.2 Hospital.

Table S5: Ablation experiments of lesion classification on external test cohorts.

	Without Phase-interaction			With Phase-interaction		
	ZZH	QZH	NBH	ZZH	QZH	NBH
Binary						
AUC (95%CI)	0.983 (0.976-0.990)	0.983 (0.974-0.991)	0.979 (0.970-0.988)	0.989 (0.983-0.994)	0.977 (0.963-0.988)	0.981 (0.972-0.989)
Accuracy (95%CI)	0.936 (0.919-0.951)	0.920 (0.900-0.940)	0.877 (0.851-0.902)	0.946 (0.930-0.961)	0.922 (0.901-0.943)	0.917 (0.894-0.937)
Precision (95%CI)	0.856 (0.809-0.897)	0.746 (0.678-0.808)	0.795 (0.748-0.838)	0.902 (0.861-0.941)	0.761 (0.699-0.824)	0.868 (0.828-0.905)
Sensitivity (95%CI)	0.929 (0.895-0.961)	0.959 (0.926-0.987)	0.974 (0.954-0.992)	0.906 (0.867-0.943)	0.932 (0.887-0.968)	0.959 (0.932-0.981)
Specificity (95%CI)	0.939 (0.919-0.958)	0.910 (0.885-0.932)	0.801 (0.757-0.844)	0.962 (0.945-0.976)	0.919 (0.894-0.941)	0.884 (0.848-0.918)
Seven-category						
AUC (95%CI)	0.958 (0.923-0.981)	0.948 (0.932-0.963)	0.951 (0.920-0.977)	0.973 (0.960-0.985)	0.970 (0.958-0.981)	0.958 (0.929-0.980)
Accuracy (95%CI)	0.868 (0.845-0.891)	0.826 (0.798-0.854)	0.801 (0.769-0.834)	0.872 (0.848-0.895)	0.856 (0.831-0.881)	0.828 (0.798-0.859)

AUC=area under the receiver operating characteristic curve.

ZZH=Zhangzhou Hospital; QZH=Quzhou People's Hospital; NBH=Ningbo No.2 Hospital.

Table S6: Ablation experiments of lesion detection on different cohorts.

3DCSWin		Internal validation	External test cohorts			Prospective cohort
			ZZH	QZH	NBH	
×	Recall	0.919	0.957	0.971	0.924	0.945
	≥1	0.931	0.967	0.984	0.932	0.950
	<1	0.862	0.907	0.942	0.878	0.922
√	Recall	0.930	0.963	0.973	0.928	0.951
	≥1	0.938	0.968	0.982	0.933	0.951
	<1	0.893	0.937	0.952	0.898	0.948
	Slice					
	thickness	0.932	-	-	-	-
	[2.5-5mm)	0.940	0.963	0.973	0.928	0.955
	[5.0-7mm)	0.907	-	-	-	0.92
	[7.0-10mm)					
	mAs					
	[0-100)	0.929	0.937	0.971	0.925	0.954
	[100,200)	0.932	0.969	0.979	0.936	0.949
	[200,500)	0.962	0.965	0.970	0.936	0.953

ZZH=Zhangzhou Hospital; QZH=Quzhou People's Hospital; NBH=Ningbo No.2 Hospital.

Table S7: Manufactures of CT scans.

	No.of patients	TOSHIBA	SIEMENS	Philips	GE	UIH
Total	12,610	319	8,055	332	3,631	273
Huzhou Central Hospital	51	43	3	5	0	0
Jinhua Municipal Central Hospital	85	0	2	83	0	0
Sir Run Run Shaw Hospital	8,291	31	5,832	10	2,172	246
Zhejiang Provincial People's Hospital	361	1	345	0	15	0
Zhejiang Cancer Hospital	332	0	228	1	103	0
Taizhou Municipal Central Hospital	156	0	0	0	156	0
The First People's Hospital of Wenling	63	14	0	0	30	19
Yinzhou People's Hospital	101	0	4	96	0	1
The Second Affiliated Hospital of Zhejiang University School of Medicine	103	13	82	0	7	1
Shaoxing People's Hospital	48	0	0	29	19	0
Ningbo No.2 Hospital	656	0	650	0	0	6
Zhangzhou Municipal Hospital of Fujian Province	844	0	20	0	824	0
Quzhou People's Hospital	529	217	273	39	0	0
Ningbo First Hospital	126	0	126	0	0	0
The First Hospital of Jiaxing	470	0	290	0	180	0
Lishui People's Hospital	65	0	40	0	25	0
The Second Hospital of Jiaxing	260	0	160	0	100	0
Jinhua Guangfu Hospital	69	0	0	69	0	0
