# Supplementary material for: A multicenter clinical AI system study for detection and diagnosis of focal liver lesions
Source: Nat Commun. 2024 Feb 7;15:1131. doi: 10.1038/s41467-024-45325-9 (PMC10850133; doi:10.1038/s41467-024-45325-9)
Supplement: Supplementary file 2 — Reporting Summary [file 41467_2024_45325_MOESM2_ESM.pdf]

Corresponding author(s): Xiujun Cai

Lastupdated by author(s): Xiujun Cai

Reporting Summary

Nature Portfolio wishes to improve the reproducibility of the work that we publish. This form provides structure for consistency and transparency in reporting. For further information on Nature Portfolio policies, see our Editorial Policies and the Editorial Policy Checklist.

Pleasedonotcompleteanyfieldwith"notapplicable"orn/a.Refertothehelptextforwhattexttouseifanitemisnotrelevanttoyourstudy.For final submission: please carefully check your responses for accuracy; you will not be able to make changes later.

Statistics

For all statistical analyses, confirm that the following items are present in the figure legend, table legend, main text, or Methods section.

n/a	Confirmed	
☐	☒	The exact sample size (n) for each experimental group/condition, given as a discrete number and unit of measurement
☐	☒	A statement on whether measurements were taken from distinct samples or whether the same sample was measured repeatedly
☐	☒	The statistical test(s) used AND whether they are one- or two-sided<br>*Only common tests should be described solely by name; describe more complex techniques in the Methods section.*
☐	☒	A description of all covariates tested
☐	☒	A description of any assumptions or corrections, such as tests of normality and adjustment for multiple comparisons
☐	☒	A full description of the statistical parameters including central tendency (e.g. means) or other basic estimates (e.g.regressioncoefficient) AND variation (e.g. standard deviation) or associated estimates of uncertainty (e.g. confidence intervals)
☐	☒	For null hypothesis testing, the test statistic (e.g. F,t,r) with confidence intervals, effect sizes, degrees of freedom and Pvalue noted *Give P values as exact values whenever suitable.*
☐	☒	For Bayesian analysis, information on the choice of priors and Markov chain Monte Carlo settings
☐	☒	For hierarchical and complex designs, identification of the appropriate level for tests and full reporting of outcomes
☒	☐	Estimates of effect sizes (e.g. Cohen's d, Pearson's r), indicating how they were calculated

Our web collection onstatistics for biologistscontains articles on many of the points above.

Software and code

Policy information about availability of computer code

Data collection	No software was used for data collection.
Data analysis	The architecture of our system is an integration of innovative technologies, including an enhanced Faster R-CNN with the CSwin Transformer for feature extraction, a U-Net augmented with the scSE attention mechanism, and DenseNet. For transparency and reproducibility, the source code and models for each component are available through open-source platforms. The respective repositories are as follows: Faster R-CNN is hosted at https://github.com/open-mmlab/mmdetection; CSwin Transformer can be found at https://github.com/microsoft/CSWin-Transformer; U-Net is available at https://github.com/milesial/Pytorch-UNet; the scSE Attention Unit is located at https://gitcode.net/mirrors/shanglianlm0525/pytorch-networks/-/blob/master/Attention/SEvariants.py; and DenseNet can be accessed at https://github.com/xmuyzz/3D-CNN-PyTorch/blob/master/models/DenseNet.py.

For manuscripts utilizing custom algorithms or software that are central to the research but not yet described in published literature, software must be made available to editors andreviewers. We strongly encourage code deposition in a community repository (e.g. GitHub). See the Nature Portfolio guidelines for submitting code & softwarefor further information.

Data

Policy information about availability of data

All manuscripts must include a data availability statement. This statement should provide the following information, where applicable:

- Accession codes, unique identifiers, or web links for publicly available datasets
- A description of any restrictions on data availability
- For clinical datasets or third party data, please ensure that the statement adheres to our policy.

All analytical data underpinning the findings of this study are incorporated within this paper in the designated Source Data files (Source_data_Figure_3.xlsx to Source_data_Figure_7.xlsx and Source_data_Figure_S1.xlsx to Source_data_Figure_S3.xlsx). The raw imaging and clinical datasets are subject to restricted access to mitigate the risk of potential misuse, despite being anonymized. These datasets can be obtained from the corresponding author upon reasonable request.

Research involving human participants, their data, or biological material

Policy information about studies with human participants or human data. See also policy information about sex, gender (identity/presentation), and sexual orientation and race, ethnicity and racism.

Reporting on sex and gender	The design of this study took into account both sex and/or gender, with these attributes determined based onself-reporting at the time of patient enrollment.
Reporting on race, ethnicity, or other socially relevant groupings	This study does not involve any socially constructed or socially relevant categorization variable(s), including but not limited to race, ethnicity, or other social relevant groupings.
Population characteristics	This study recruited participants aged 14 years and older who underwent enhanced CT scans for focal liver lesions (FLLs). Patients who had undergone any form of treatment for FLLs prior to the contrast enhanced CT scan were excluded. This includes those who received surgery, transcatheter arterial chemoembolization (TACE), radiofrequency ablation, chemotherapy, radiotherapy, targeted drug therapy or immunotherapy.
Recruitment	In our study, we enrolled participants aged 14 years and above who underwent enhanced CT scans for diagnosing focal liver lesions (FLLs). Each participant underwent a comprehensive triple-phase CT scan, which included non-contrast, arterial, and portal venous phases. We also collected key clinical data, encompassing basic demographics like age and gender, along with relevant medical history such as hepatitis, cirrhosis, cholangiolithiasis, and extra-hepatic tumors. This information was sourced from self-reports and Electronic Medical Records (EMRs) at the time of enrollment. To mitigate potential biases and enhance the representativeness of our training dataset, we diversified our sample collection by including participants from 18 different hospitals. This approach ensured a broad spectrum of demographic and clinical variations, contributing to the robustness and generalizability of our findings.
Ethics oversight	This study has received approval from the Institutional Review Board (IRB) of Sir Run Run Shaw Hospital (SRRSH) and was carried out in adherence to the Declaration of Helsinki. Additionally, the prospective component of this study is officially registered with the Chinese Clinical Trial Registry, under the identifier ChiCTR2100045278 (accessible at [https://www.chictr.org.cn/showproj.html?proj=124700], registration date: April 10, 2021). In parallel, all 17 collaborating institutions obtained requisite IRB approvals for their participation in the retrospective aspects of the study. Owing to its non-invasive methodology, the IRB granted a waiver for the informed consent requirement.

Note that full information on the approval of the study protocol must also be provided in the manuscript.

Field-specific reporting

Please select the one below that is the best fit for your research. If you are not sure, read the appropriate sections before making your selection.

☒ Life sciences ☐ Behavioural & social sciences ☐ Ecological, evolutionary & environmental science

For a reference copy of the document with all sections, see nature.com/documents/nr-reporting-summary-flat.pdf

Life sciences study design

All studies must disclose on these points even when the disclosure is negative.

Sample size	This study included retrospective data from 11,385 patients managed in18hospitals in China between January 1st, 2010 and June 30th 2020, to develop and validate the proposed LiAIDS. In addition, a prospective study further included 1,225 patients treated at SRRSH between July1st, 2020 and June 30th, 2021. Furthermore, 13,192 consecutive patients admitted to SRRSH between May 1st 2022 and August 31th 2022were collected for a study of patient triage. As a common rule of thumb in traditional statistical studies, it's usually recommended to have at least 30 instances for each class in the test set. Although somewhat arbitrary, this guideline typically strikes a decent balance between achieving statistical significance and managing computational expense. In our study, we use a large number of samples from different centers and the counts for each of the 7 lesion classes all exceed this threshold, as detailed in Table 1. Therefore, we are confident that the number of lesions in each class is ample for reliable model assessment. As for our non-inferiority study, the 183 pathologically confirmed patients exceeded our initial sample size calculation for the trial. This estimation, based on a non-inferiority margin (delta, δ) of 0.1, a significance level (alpha, α) of 0.05, a power (1-beta, 1-β) of 0.8, and an expected efficacy accuracy of 0.92, had anticipated a need for 173 participants. Therefore, our study was adequately powered as the actual number of recruited participants surpassed the calculated necessity.
Data exclusions	Exclusion criteria were: (1) patients who received any form of treatment for FLLs prior to the contrast-enhanced CT scan, including surgery, transcatheter arterial chemoembolization (TACE), radiofrequency ablation, chemotherapy, radiotherapy, targeted drug therapy, and immunotherapy; (2) patients who had a clinical diagnosis of malignant lesions but lacked pathological confirmation; (3) benign cases that lacked both a histopathological report and a consensus agreement; (4) cases with compromised CT image quality due to reasons including patient movement, incorrect positioning, presence of metallic objects, or equipment malfunctions; and (5) cases that lacked essential clinical information, including basic patient data (for example age and gender) and relevant medical history (such as hepatitis, cirrhosis, cholangiolithiasis, and extra-hepatic tumors).
Replication	The data are available from the corresponding author upon reasonable request. The architecture of our system is an integration of innovative technologies, including an enhanced Faster R-CNN with the CSwin Transformer for feature extraction, a U-Net augmented with the scSE attention mechanism, and DenseNet. For transparency and reproducibility, the source code and models for each component are available through open-source platforms. The respective repositories are as follows: Faster R-CNN is hosted at https://github.com/open-mmlab/mmdetection; CSwin Transformer can be found at https://github.com/microsoft/CSWin-Transformer; U-Net is available at https://github.com/milesial/Pytorch-UNet; the scSE Attention Unit is located at https://gitcode.net/mirrors/shanglianlm0525/pytorch-networks/-/blob/master/Attention/SEvariants.py; and DenseNet can be accessed at https://github.com/xmuyzz/3D-CNN-PyTorch/blob/master/models/DenseNet.py.
Randomization	In this study, the retrospective dataset was partitioned into an internal cohort comprising data from 15 hospitals and three independent external cohorts, consisting of data from the remaining 3 hospitals, respectively. Participants in the internal cohort were randomly divided into internal training and validation cohorts at a 4:1 ratio. For the purpose of external validation, we specifically selected the three largest sites, excluding the largest SRRSH, to form our external validation cohorts. This approach guarantees a comprehensive performance assessment over a wide range of data, thereby capturing a greater diversity of patient scenarios.
Blinding	Yes, the investigators were blinded to group allocation during data collection and analysis.

Reporting for specific materials, systems and methods

We require information from authors about some types of materials, experimental systems and methods used in many studies. Here, indicate whether each material, system or method listed is relevant to your study. If you are not sure if a list item applies to your research, read the appropriate section before selecting a response.

Materials & experimental systems

n/a	Involved in the study
☒	☐ Antibodies
☒	☐ Eukaryotic cell lines
☒	☐ Palaeontology and archaeology
☒	☐ Animals and other organisms
☐	☒ Clinical data
☒	☐ Dual use research of concern
☒	☐ Plants

Method

n/a	Involved in the study
☒	☐ ChIP-seq
☒	☐ Flow cytometry
☒	☐ MRI-based neuroimaging

Clinical data

Policy information about clinical studies

All manuscripts should comply with the ICMJE guidelines for publication of clinical research and a completed CONSORT checklist must be included with all submissions.

Clinical trial registration

This study has received approval from the Institutional Review Board (IRB) of Sir Run Run Shaw Hospital (SRRSH) and was carried out in adherence to the Declaration of Helsinki. The prospective component of this study is officially registered with the Chinese Clinical Trial Registry, under the identifier ChiCTR2100045278 (accessible at [https://www.chictr.org.cn/showproj.html?proj=124700], registration date: April 10, 2021).

Study protocol

The study design is described in our manuscript (method part).

Data collection

Our study utilized retrospective data from 11,385 patients across 18 hospitals in China, collected between January 1, 2010, and June 30, 2020. This dataset was used for the development and validation of the LiAIDS (Liver Artificial Intelligence Diagnostic System). A subsequent phase of the study, from July 1, 2020, to June 30, 2021, involved a prospective analysis of 1,225 patients at Sir Run Run Shaw Hospital (SRRSH). Furthermore, an additional data collection phase occurred between May 1, 2022, and August 31, 2022, at SRRSH. This phase focused on triage analysis and included data from 13,192 consecutive patient admissions. The study's dataset represents consecutively enrolled patients from all the specified time periods at the 18 participating hospitals. For comprehensive details about these hospitals and the corresponding data, please refer to Table S7 and Figure S3 in the supplementary materials.

Outcomes

We employed Receiver Operating Characteristic (ROC) curves to evaluate our model's diagnostic performance. These curves were generated by varying the threshold for predicted probability and plotting the True Positive Rate (TPR, or sensitivity) against the False Positive Rate (FPR, 1-specificity). A high Area Under the Curve (AUC) indicates superior diagnostic capability. In our analysis, the AUC and confusion matrix were the primary metrics, providing comprehensive insights into model efficacy across different lesion types and addressing class imbalance. Additionally, we considered the following secondary metrics for performance evaluation: Accuracy, calculated as (TP+TN)/(TP+TN+FP+FN), Sensitivity, defined as (TP)/(TP+FN), Specificity, determined as (TN)/(TN+FP), and Precision, computed as (TP)/(TP+FP). Here, TP denotes True Positives, TN denotes True Negatives, FP denotes False Positives, and FN denotes False Negatives. For the comparison between AI and radiologists, the F1-score, the harmonic mean of sensitivity and precision was also used as this metric offers a balanced view of both sensitivity and precision in a single measure. The F1-score is calculated as 2 * (sensitivity * precision) / (sensitivity + precision). All statistical analyses employed two-tailed tests, with p-values of 0.05 or lower deemed significant. These analyses were conducted using Python, version 3.7.6.

This checklist template is licensed under a Creative Commons Attribution 4.0 International License, which permits use, sharing, adaptation, distribution and reproduction in any medium or format, as long as you give appropriate credit to the original author(s) and the source, provide a link to the Creative Commons license, and indicate if changes were made. The images or other third party material in this article are included in the article's Creative Commons license, unless indicated otherwise in a credit line to the material. If material is not included in the article's Creative Commons license and your intended use is not permitted by statutory regulation or exceeds the permitted use, you will need to obtain permission directly from the copyright holder. To view a copy of this license, visit http://creativecommons.org/licenses/by/4.0/
